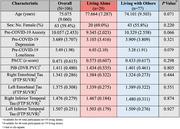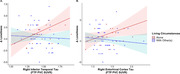# Living alone, medial temporal tau pathology, and loneliness in cognitively normal older adults during the COVID‐19 pandemic

**DOI:** 10.1002/alz.089624

**Published:** 2025-01-09

**Authors:** Benjamin S Zide, Geoffroy Pierre Gagliardi, Heidi I.L. Jacobs, Jennifer R. Gatchel, Yakeel T. Quiroz, Gad A Marshall, Keith A Johnson, Reisa A Sperling, Patrizia Vannini, Nancy J Donovan

**Affiliations:** ^1^ Brigham and Women’s Hospital, Boston, MA USA; ^2^ Massachusetts General Hospital, Boston, MA USA; ^3^ Maastricht University, Maastricht Netherlands; ^4^ Harvard Medical School, Boston, MA USA; ^5^ Department of Psychiatry, Massachusetts General Hospital, Harvard Medical School, Boston, MA USA

## Abstract

**Background:**

Social restrictions during the COVID‐19 pandemic raised acute concerns about the impacts of loneliness on older adults’ well‐being, particularly for those who live alone. Loneliness is a perceived state of isolation from others that is only partly determined by quantities of social ties and interactions. Drawing a subsample from the Harvard Aging Brain Study, we measured self‐reported loneliness in older adults living alone and those living with others during the pandemic. We leveraged pre‐COVID‐19 loneliness and neuroimaging data to evaluate whether Alzheimer’s disease (AD) pathology contributed to rising loneliness levels in community dwelling older adults living alone during the pandemic.

**Method:**

One‐hundred and six cognitively unimpaired older adults underwent clinical assessments of self‐reported loneliness (UCLA Loneliness scale), cognitive performance (Preclinical Alzheimer’s Cognitive Composite [PACC]), depression (Geriatric Depression Scale), anxiety (Hospital Anxiety and Depression Scale), PiB‐PET measurements of cortical Aβ and FTP‐PET measurements of entorhinal cortex (EC) and inferior temporal (IT) tau prior to the pandemic. In May 2020, participants completed online surveys to determine living circumstances (alone vs. with others) and to re‐assess loneliness. Linear regressions with backward elimination estimated the interactive effect of living alone and either PiB, unilateral EC FTP, IT FTP, or PACC with change in loneliness (Δ Loneliness), adjusting for age, sex, time since pre‐COVID‐19 assessment, and pre‐COVID‐19 depression, anxiety, and loneliness.

**Result:**

Table 1 presents sample characteristics. Before May 2020, pre‐COVID‐19 loneliness levels did not differ between participants living alone or those living with others (t [103] = ‐1.77, P = 0.08). Participants living alone during COVID‐19 reported greater increases in loneliness compared to those living with others, adjusting for covariates (β = 1.22, P = 0.024). Greater right EC and IT FTP binding in individuals living alone was associated with greater increases in loneliness (for living alone*EC FTP: β = ‐4.31, P = 0.044; R2 = 0.18; for living alone*IT FTP: β = 7.45, P = 0.019; R2 = 0.20; Figure 1). Interactions of PiB and PACC with living alone predicting change in loneliness were not significant.

**Conclusion:**

Greater tau pathology compounded the association of living alone with increases in loneliness in cognitively unimpaired older adults during the COVID‐19 pandemic. AD pathological brain changes may be an unrecognized factor which contributes to late‐life loneliness.